# Special Issue “Gut Microbioma Structure and Functions in Human Health and Disease 2.0”: Editorial

**DOI:** 10.3390/microorganisms11030740

**Published:** 2023-03-14

**Authors:** Francesco Di Pierro

**Affiliations:** Scientific Department, Velleja Research, 20124 Milan, Italy; f.dipierro@vellejaresearch.com

Recent technical advances in the analysis of human colonic bacterial consortia have led to a considerable explosion of research on the gut microbiota [1]. These advances have also generated intense interest among those clinicians motivated to apply these results to their patients [2]. As complex as it is translating the results of such investigations into something useful from a clinical perspective, we are however rather certain that there is a noticeable decrease in the alpha biodiversity of microbiota in people affected by certain pathologies (obesity, diabetes, cancer, immune diseases, etc.) [3] and that a strong correlation does exist between a phylum (e.g., Proteobacteria) or taxon (e.g., *Fusobacterium*) with these diseases [4,5]. Similarly, in these contexts, the positive role played by some genera (e.g., *Akkermansia* or *Faecalibacterium*) cannot be denied [6]. Despite this advance in knowledge, much remains unclear. With this Special Issue “Gut Microbiome Structure and Functions in Human Health and Disease 2.0”, some authors have tried to propose their perspectives on this topic.

Various parameters obtainable via the analysis of the human gut microbiota have been investigated to develop applications for clinical medicine. Among these parameters, the enterotypes, Firmicutes-to-Bacteroidetes ratio, *Prevotella*-to-*Bacteroides* ratio, Gram-positive-to-Gram-negative ratio and *Fusobacterium*-to-*Faecalibacterium* ratio are the most widely used [7,8]. For example, the enterotype B2, especially in the presence of high calprotectin and C reactive protein, has been strongly correlated with primary sclerosing cholangitis, Crohn’s disease and, to a lesser extent, ulcerative colitis [9]. Characterized by a low proportion of butyrate producers, enterotype B2 has also been associated with depression [10]. The *Bacteroides*-dominant enterotype has been correlated with liver inflammation, metabolic syndrome, type 2 diabetes, colon cancer and celiac disease, while the *Prevotella*-dominant enterotype is associated with rheumatoid arthritis and hypertension and the *Ruminococcus*-dominant enterotype with cardiovascular disease and cognitive decline [11]. The Firmicutes/Bacteroidetes (F/B) ratio was initially discovered and proposed by Ley et al. and has been frequently cited in the scientific literature as a measure of obesity [12]; however, it has been recently challenged due to several contradictory results [13]. These discrepancies are likely due to the impact of ignoring lifestyle-associated confounders and oversimplification as Firmicutes are often considered as a group of bacteria possessing the same types of characteristics, which is incorrect [6]. From a caloric perspective, it has been calculated that with a 20% increase in Firmicutes, the gut microbiota can “capture” an additional 150 kcal in the colon [14]. Thus, the F/B ratio cannot be considered a marker of obesity, but it could be used to evaluate a predisposition to lose “colonic” calories. In addition, in longitudinal studies, it could be used to follow the changes in the gut microbiota during a low-calorie diet, since this ratio correlates with the performance of colonic microbiota in regard to the exhaustive absorption of calories. Less investigated than the F/B ratio, the Prevotella/Bacteroides ratio has been proposed to predict the ease with which people can lose weight when subjected to a low-calorie, fibre-rich diet. Finally, the Gram-positive/Gram-negative ratio has been proposed to identify patients with severe fragility, and the Fusobacterium/Faecalibacterium ratio has been proposed for diagnosing patients who are at a high risk of developing colon cancer [7].

The gut microbiota of people of different nationalities and religions are strongly influenced by food preferences. After confirming the well-known different microbiota profiles according to the different countries, Syromyatnikov and his colleagues investigated the possible role played by different religions. They demonstrated that among Buddhists and Muslims, the Prevotella enterotype dominates the gut microbiome, while in people of other religions, including Christians, the Bacteroides enterotype is the most common [15]. This is mainly due to the food components that characterize the different diets, especially based on their fibre content, which was also confirmed by the review proposed by Bertuccioli et al. [16].

Last but not the least, the study of Baltazar-Díaz et al. [17] focused on the relative presence of the OUT belonging to the *Escherichia*/*Shigella* group. They found that the gut’s short-chain fatty acids content and the associated metabolic pathways could be used to better characterize the intestinal dysbiosis typically observed in patients with decompensated alcoholic cirrhosis [17].
